# Associations of Bowel Movement Frequency with Risk of Cardiovascular Disease and Mortality among US Women

**DOI:** 10.1038/srep33005

**Published:** 2016-09-06

**Authors:** Wenjie Ma, Yanping Li, Yoriko Heianza, Kyle D. Staller, Andrew T. Chan, Eric B. Rimm, Kathryn M. Rexrode, Lu Qi

**Affiliations:** 1 Department of Epidemiology, Harvard T.H. Chan School of Public Health, Boston, MA 02115, USA; 2 Department of Nutrition, Harvard T.H. Chan School of Public Health, Boston, MA 02115, USA; 3 Department of Epidemiology, School of Public Health and Tropical Medicine, Tulane University, New Orleans, Louisiana 70112, USA; 4 Division of Gastroenterology, Massachusetts General Hospital and Harvard Medical School, Boston, MA 02114, USA; 5 Clinical and Translational Epidemiology Unit, Massachusetts General Hospital, Boston, MA 02114, USA; 6 Channing Division of Network Medicine, Department of Medicine, Brigham and Women’s Hospital and Harvard Medical School, Boston, MA 02115, USA; 7 Division of Preventive Medicine, Department of Medicine, Brigham and Women’s Hospital and Harvard Medical School, Boston, MA 02115, USA

## Abstract

Emerging evidence suggests a potential impact of gastrointestinal function on cardiometabolic risk. Abnormal bowel movements have been related to various cardiovascular risk factors such as dyslipidemia, hypertension, diabetes, and altered metabolism of bile acids and gut microbiota. However, little is known about whether bowel movement frequency affects risk of cardiovascular disease (CVD) and mortality. In the Nurses’ Health Study, bowel movement frequency was self-reported in 1982 by 86,289 women free from CVD and cancer. During up to 30 years of follow-up, we documented 7,628 incident CVD cases and 21,084 deaths. After adjustment for dietary intake, lifestyle, medication use, and other risk factors, as compared with women with daily bowel movement, having bowel movements more than once daily was significantly associated with increased risk of CVD (hazard ratio [HR]: 1.13; 95% confidence interval [CI]: 1.05–1.21), total mortality (HR: 1.17; 95% CI: 1.12–1.22), and cardiovascular mortality (HR: 1.17; 95% CI: 1.07–1.28). With further adjustment for body mass index and diabetes status, the association with total mortality remained significant (HR: 1.10; 95% CI: 1.06–1.15), whereas the associations with incident CVD and cardiovascular mortality were no longer significant. Our results suggest increased bowel movement frequency is a potential risk factor for premature mortality.

The function of the gastrointestinal (GI) tract has been thought to have little substantive impact on cardiovascular health. However, recently emerging evidence suggests that GI function may play a greater role than previously thought^1^. Several digestive diseases, such as inflammatory bowel disease and gallstone disease, have been associated with an increased risk of cardiovascular disease (CVD)^2^,^3^,^4^,^5^ as well as various cardiometabolic risk factors such as obesity, inflammation, dyslipidemia, and insulin resistance^6^,^7^. In addition, disorders of GI system are closely related to abundance, type, and metabolism of gut microbiota^8^,^9^,^10^,^11^,^12^, a vast and diverse microbial ecosystem in the human intestine with known impact on risk of CVD and diabetes^13^,^14^,^15^,^16^.

Bowel movement is the endpoint of digestion, and changes in bowel movement frequency represents one of the major outward characteristics of functional bowel disorders^17^. In previous studies, abnormal bowel movement frequency occurring in irritable bowel syndrome and functional constipation has been related to a variety of cardiovascular risk factors including elevated circulating levels of cholesterol and triglycerides, hypertension, diabetes, and altered bile acid metabolism^18^,^19^,^20^,^21^,^22^,^23^,^24^,^25^. However, to date, very few studies have assessed the association of bowel movement frequency with CVD and mortality in prospective settings^19^,^26^,^27^.

In the present study, we prospectively assessed the associations of bowel movement frequency with risk of incident CVD and mortality among generally healthy US women in the Nurses’ Health Study (NHS). Because bowel movement frequency is affected by dietary and lifestyle factors, we particularly examined whether these factors modified the associations.

## Results

### Baseline characteristics of study participants

Distributions of demographics and lifestyle factors at baseline across categories of bowel movement frequency are shown in Table 1. As compared to women with bowel movement frequency of once daily, those with more frequent bowel movements (i.e., more than once daily) had higher BMI and total energy intake, and were also more likely to have hypertension, hypercholesterolemia, diabetes, ulcerative colitis, cholecystectomy and use multivitamin, aspirin, other nonsteroidal anti-inflammatory drugs, thiazide diuretics, and thyroid hormone. Women with infrequent bowel movements (e.g., every 3 days or less) were younger and had lower BMI, physical activity level, alcohol intake, and total energy intake, as well as an increased likelihood to use aspirin but a reduced likelihood to use multivitamin. There was also an obvious correlation between bowel movement frequency and laxative use, with a significantly higher prevalence of laxative use in a weekly to daily manner among participants with infrequent bowel movements. Notably, energy-adjusted total fiber intake did not substantially differ across categories of bowel movement frequency. The baseline characteristics of participants who answered the questions on bowel movement frequency were fundamentally similar with those who did not answer these questions (data not shown).

We also combined the categories of non-daily bowel movements (more than once daily or less than once daily) and compared these participants’ characteristics to those with bowel movement frequency of once daily (Supplemental Table 1). Most lifestyle factors and dietary intakes were significantly different between the two groups. For example, women with non-daily bowel movements had lower physical activity level and alcohol intake; and they were more likely to have history of diabetes, ulcerative colitis, and cholecystectomy and use laxatives in a weekly to daily manner. These associations remained in the multivariate analysis by controlling for major demographic, lifestyle, and dietary factors simultaneously in the model (Supplemental Table 2).

### Associations of bowel movement frequency with risk of incident CVD and mortality

A total of 7,628 incident CVD cases (3,817 CHD cases and 3,901 stroke cases) occurred during 30 years of follow-up (Table 2). In age-adjusted analyses, having a bowel movement more than once daily was significantly associated with an increased risk of developing CVD, CHD, and stroke. As compared to women with bowel movement frequency of once daily (reference), the HRs for those with more than once daily were 1.25 (95% CI: 1.16, 1.33; P for trend <0.001) for incident CVD, 1.22 (95% CI: 1.11, 1.34; P for trend <0.001) for CHD, and 1.26 (95% CI: 1.15, 1.39; P for trend <0.001) for stroke. After adjustment for potential confounders including demographics, lifestyle, and dietary factors, the associations were attenuated but remained significant. With additional adjustment for factors that were considered to be either confounders or mediators of the association including BMI and diabetes status at baseline, the associations of more frequent bowel movements with stroke (HR: 1.10; 95% CI: 1.00, 1.22; P for trend = 0.03) became borderline significant, whereas the association with CVD (HR: 1.05; 95% CI: 0.98, 1.13; P for trend = 0.04) and CHD (HR: 1.00; 95% CI: 0.90, 1.10; P for trend = 0.57) were no longer significant. Infrequent bowel movements (e.g., every 3 days or less) were not significantly associated with any of these disease outcomes.

We documented 21,084 all-cause deaths including 4,491 deaths due to CVD (Table 3). As compared to women with daily bowel movements, more frequent bowel movements were also associated with increased risk of total mortality (HR: 1.25; 95% CI: 1.20, 1.30; P for trend <0.001) and cardiovascular mortality (HR: 1.32; 95% CI: 1.21, 1.44; P for trend < 0.001) in the age-adjusted model. The multivariable-adjusted HRs for women with more than once daily were 1.17 (95% CI: 1.12, 1.22; P for trend <0.001) for total mortality and 1.17 (95% CI: 1.07, 1.28; P for trend <0.001) for cardiovascular mortality. After further adjusting for BMI and diabetes, the association with total mortality was attenuated but remained significant (HR: 1.10; 95% CI: 1.06, 1.15; P for trend <0.001), whereas the association with cardiovascular mortality was no longer significant (HR: 1.05; 95% CI: 0.96, 1.15; P for trend = 0.005). Infrequent bowel movements were not significantly related to total mortality or cardiovascular mortality.

Findings were similar in sensitivity analyses by excluding cases in the first 4 years (Supplemental Table 3). The associations became even stronger when we adjusted for updated covariates to account for their variations over time (Supplemental Table 4). Compared to women with bowel movement frequency of once daily, the adjusted HRs for those with more than once daily in the final models were 1.11 (95% CI: 1.04, 1.19; P for trend <0.001) for CVD, 1.16 (95% CI: 1.12, 1.21; P for trend <0.001) for total mortality, and 1.17 (95% CI: 1.08, 1.28; P for trend <0.001) for cardiovascular mortality. Additionally, when laxative use was jointly considered, compared to participants who had daily bowel movements and never used laxatives, the multivariable-adjusted HR (95% CI) for those who had both infrequent bowel movements and weekly to daily laxative use was 0.81 (0.64, 1.02) for incident CVD, 0.88 (0.77, 1.01) for total mortality, and 0.75 (0.54, 1.04) for cardiovascular mortality.

### Stratified analysis by dietary and lifestyle factors

In stratified analyses of bowel movement frequency and CVD according to baseline characteristics including age, BMI, smoking status, alcohol intake, physical activity, hypertension, diabetes, laxative use, total fiber intake, red meat intake, total energy intake, and AHEI score (Table 4), significant interactions were observed of bowel movement frequency with age, red meat intake, and AHEI score on CVD risk (P for interaction = 0.01, 0.005, and 0.03, respectively). Compared to women with bowel movement frequency of once daily, the increased risk associated with more frequent bowel movements was only seen among those with younger age (HR: 1.25; 95% CI: 1.09, 1.43), those with lower red meat intake (HR: 1.20; 95% CI: 1.06, 1.35), or those with higher AHEI score (HR: 1.15; 95% CI: 1.03, 1.28), but not among the rest of the participants. The associations were not significantly modified by any of the other factors.

**Figure 1:**
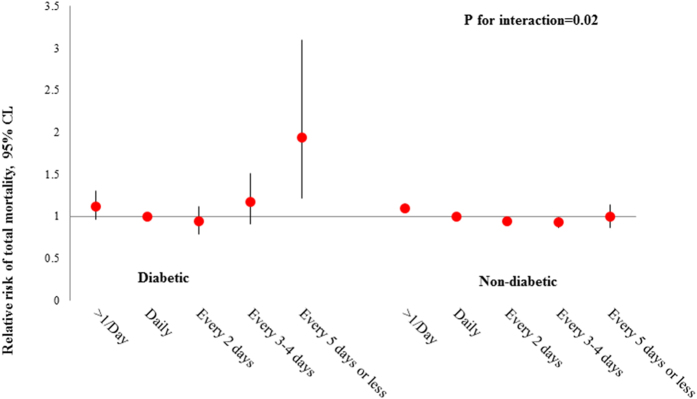
Association between bowel movement frequency and risk of total mortality according to baseline diabetes status. Models were adjusted for the same covariates as shown in Model 4, Table 2.

**Figure 2:**
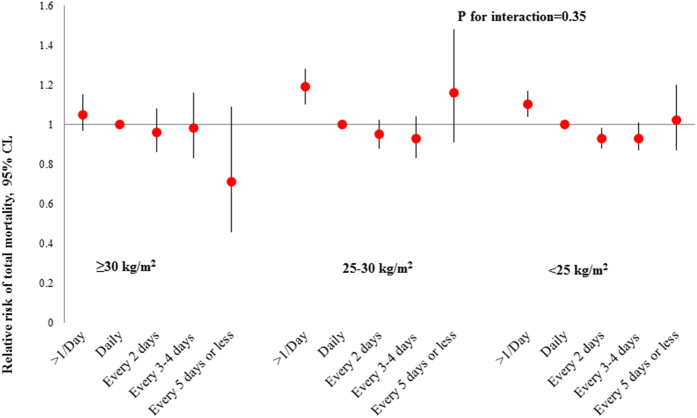
Association between bowel movement frequency and risk of total mortality according to baseline body mass index. Models were adjusted for the same covariates as shown in Model 4, Table 2.

In addition, we found that the relation of bowel movement frequency with total mortality was significantly modified by diabetes status (P for interaction = 0.02; Fig. 1). Compared to bowel movement frequency of once daily, extremely infrequent bowel movements (i.e., every 5 days or less) were associated with an increased risk of total mortality among women who had history of diabetes (HR: 1.94; 95% CI: 1.21, 3.10), but not among others (HR: 1.00; 95% CI: 0.87, 1.14). When stratified by BMI (Fig. 2), more frequent bowel movements were associated with an increased risk of total mortality among participants who were overweight (HR: 1.19; 95% CI: 1.10, 1.28) or with a BMI < 25 kg/m^2^ (HR: 1.10; 95% CI: 1.04, 1.17), but not among obese participants (HR: 1.05; 95% CI: 0.97, 1.15; P for interaction = 0.35). The relations of bowel movement frequency with total mortality were not significantly modified by any of the other baseline characteristics (data not shown).

## Discussion

In this large cohort of US women, increased bowel movement frequency was associated with elevated cardiovascular risk, which might be partly mediated by BMI and diabetes. Compared to women with daily bowel movements, those with more frequent bowel movements also showed a modest increase in the risk of total mortality independent of traditional risk factors. In addition, the association of bowel movement frequency with incident CVD was modified by age, red meat intake, and dietary quality, while the association with total mortality was modified by diabetes status.

At baseline, the prevalence of major cardiovascular risk factors including BMI and baseline history of hypertension, hypercholesterolemia, and diabetes was higher in women with more frequent bowel movements. Therefore, the finding of an association of increased bowel movement frequency with elevated risk of CVD and mortality was not surprising, suggesting that higher bowel movement frequency is a marker for cardiovascular risk in middle-aged women. The associations were independent of various cardiovascular risk factors including diet, lifestyle, and medication use, but might be partly explained by BMI and diabetes. Our data suggest that increased bowel movement frequency might influence obesity^28^, diabetes^24^, and metabolic syndrome^20^ in a manner that subsequently contribute to an increased risk of CVD. Further investigations are warranted to elucidate the temporal relationship of usual bowel movement patterns with long-term weight gain and onset of metabolic disorders such as diabetes. Notably, the association between more frequent bowel movements and risk of total mortality remained significant after adjusting for these risk factors in our analysis, indicating that additional mechanisms might be involved. This independent association might be due to residual or unmeasured confounding, which was unlikely to be excluded because some variables correlated with increased bowel movement frequency and mortality risk were not controller for, such as subclinical hyperthyroidism^29^ and use of proton pump inhibitors^30^. Another potential mechanism underlying the association between increased bowel movement frequency and total mortality might be through alterations of gut microbiota, a complex community of intestinal microorganisms. Bowel movement is closely related to the abundance, type, and function of gut microbiota^10^,^11^,^12^, which interacts with host functions and has been implicated in diseases including inflammatory bowel disease, irritable bowel syndrome, and antibiotic-associated diarrhea^31^. Recently, the contributory role of gut microbiota in the development of atherosclerosis and CVD has been consistently observed in several large-scale clinical cohorts^13^,^14^,^15^. Therefore, we postulated that the relation of bowel movement with mortality might be at least partly through effects on gut microbiota.

To the best of our knowledge, very few previous studies have examined the association of bowel movement frequency with CVD or mortality^19^,^26^,^27^. In contrast to our results, severe constipation (having difficulty in bowel movements and symptom was so bothersome that usual activities could not be performed) was independently associated with a 23% higher risk of CVD in a cohort of postmenopausal women^19^. However, in that study, information about constipation was self-reported and limited to the previous 4 weeks. It has been suggested that self-reported subjective constipation is not as specific and sensitive as symptom-based criteria^32^ such as number of bowel movements or standard diagnostic criteria for GI disorders, the Rome III criteria, which defines functional constipation according to frequency, form, and passage of stool^33^; and short-term (4-week) constipation may not reflect the subject’s long-term normal bowel movement frequency. In another 2 Japanese cohorts, it was also reported that the prevalence of cardiovascular risk factors such as diabetes, stress, depression, and physical inactivity was higher in subjects with infrequent bowel movements^26^, and infrequent bowel movements were independently associated with increased risk of cardiovascular mortality^27^, but a bowel movement frequency of more than once daily was not separately assessed. In our study, among participants with bowel movement frequency of every 5 days or less, the prevalence of hypercholesterolemia and diabetes was higher, whereas BMI and hypertension prevalence were lower. Nevertheless, after multivariate adjustment, bowel movement frequency of every 5 days or less was associated with an increased risk of total mortality among a subset of population who had a history of diabetes, but not among others. These data indicate that extremely infrequent bowel movements, reflecting chronic constipation, might particularly affect high-risk populations such as those with diabetes.

Intriguingly, we found that the relation between bowel movement frequency and CVD risk was modified by age, red meat intake, and dietary quality as assessed by AHEI score. More frequent bowel movements were associated with increased CVD risk among participants who were younger, had lower red meat intake, or had higher dietary quality, whereas the association was null or inverse among the rest of participants. In addition, although the interaction was not significant, the positive association of more frequent bowel movements with CVD and total mortality was far more pronounced among overweight participants. On one hand, this observation might be explained by the increase in absolute risk by older age, higher red meat intake, or lower dietary quality among those with daily bowel movement, which attenuated the relative risk of other groups. On the other hand, the different associations might be due to potential modifications of these environmental factors on GI functions and the metabolism of gut microbiota^34^. For example, previous studies have shown red meat intake influences CVD risk, at least partly through influencing gut microbiota metabolites^35^. Obesity also affected the diversity and relative abundance of microbial population with relative increases in the Firmicutes and decreases in Bacteroidetes, which could potentially regulate energy harvest^36^,^37^. Our data provide novel support for the complex and dynamic interplay between dietary and lifestyle factors and host GI functions in the etiology of CVD.

Our study has several strengths. We used a well-established cohort with high follow-up rate and well-validated assessment of cases, which minimized selection and ascertainment biases. The prospective design and long-term follow-up reduced the potential for reverse causation. Results were similar when excluding cases in the first 4 years, further confirming the temporality of the observed association. A large sample size provided the statistical power to detect relevant associations. Comprehensive information on demographics, lifestyle habits, and diet minimized the residual confounding. Several potential limitations should be considered. First, our cohort included mostly Caucasian adults, and the results may not be generalizable to other ethnic groups. However, the relative homogeneity of the study populations in educational attainment and socioeconomic status enhanced the internal validity. Second, information on bowel movement frequency was obtained only once without updating, and changes over time could cause misclassification and potentially attenuate true associations. Finally, although we adjusted for multiple important risk factors for CVD and mortality, residual confounding might exist because of unmeasured or imprecisely measured confounders. However, the associations became stronger when we updated the covariates to account for their variations over time in sensitivity analysis, indicating that the reported effect sizes might still be underestimated.

In conclusion, we showed that compared to those with daily bowel movements, more frequent bowel movements were significantly associated with higher cardiovascular risk, which might be partly explained by BMI and diabetes. Increased bowel movement frequency was associated with a modest increase in the risk of total mortality independent of traditional risk factors. In addition, age, diet, and diabetes might modify these associations. Future investigations are warranted to verify our findings in other populations and elucidate the underlying biological mechanisms that link bowel movement frequency with risk of CVD and mortality.

## Methods

### Study population

The NHS is a prospective study of a cohort of 121,701 female registered nurses aged 30–55 years from 11 US states who were enrolled in 1976. Participants were followed with the use of biennial questionnaires with detailed reporting of medical history, lifestyle, and health practices. The follow-up rate has been greater than 90% for the cohort. The study protocol was guided by the Ethical Principles and Guidelines for the Protection of Human Subjects of Research, known as The Belmont Report, and was approved by The Institutional Review Board at Brigham and Women’s Hospital. Return of the questionnaires was considered to imply informed consent, and written consent from each participant was also collected to obtain and review medical records.

The baseline year for the current analysis was set as 1982 when information on bowel movement frequency was assessed. We excluded participants with CVD (n = 3,004) and cancer (n = 5,206) at baseline and those with missing information on bowel movement frequency (n = 15,047), leaving a total of 86,289 women for this analysis. We also compared the baseline characteristics of participants who answered the questions on bowel movement frequency with those who did not.

### Assessment of bowel movement frequency and covariates

In the 1982 questionnaire, participants were asked to report their frequency of bowel movements from 7 possible answers, including more than once daily, daily, every other day, every 3–4 days, every 5–6 days, and once a week or less. Data on demographics, medial history, and lifestyle habits have been collected by self-administered questionnaires since baseline and updated biennially. Hypertension and hypercholesterolemia were self-reported, with the validity of these reports confirmed on random sampling of medical records^38^. A supplementary questionnaire was used to confirm self-reported cases of diabetes according to established criteria^39^, and 98% of these cases were validated on comparison with medical records. Information on smoking status, menopausal status and postmenopausal hormone use, family history of myocardial infarction, multivitamin and medication use, height, and weight was obtained; self-reported weight was validated against technician-measured weight (r = 0.97)^40^. Body mass index (BMI) was calculated as weight in kilograms divided by height squared in meters. Physical activity was assessed every 2–4 years using validated questionnaires^41^. Every 4 years, usual dietary habits were assessed by means of validated semiquantitative food-frequency questionnaires that inquired about usual consumption of foods, beverages, and supplements during the previous year^42^.

### Ascertainment of outcomes

The primary endpoints for this analysis included incident CVD events and deaths that occurred after the return of the baseline questionnaire but before the end of follow-up (June 30, 2012). Incident CVD was defined as coronary heart disease (CHD) (symptomatic nonfatal myocardial infarction or fatal CHD) and stroke (nonfatal or fatal), which was identified primarily through a review of medical records, as previously described^43^. Participants (or next of kin for decreased participants) reporting a primary endpoint were asked for permission to have their medical records reviewed by physicians who were blinded to the participant’s self-reported risk factor status. Nonfatal myocardial infarction or nonfatal stroke was classified as confirmed if they met the criteria of the World Health Organization^44^ or the National Survey of Stroke. CHD and stroke events for which confirmatory information was obtained by interview or letter but no medical records were available were designated as probable events. For the analyses we included both confirmed and probable cases to maximize statistical power.

Deaths were identified from state vital statistics records and the National Death Index or reported by the families and the postal system. The underlying cause of death was assigned according to the *International Classification of Diseases, 8th Revision (ICD-8)*. Fatal CHD (*ICD-8* codes 410-412) was considered to have occurred if fatal MI was confirmed by hospital records or autopsy or if CHD was listed as the cause of death on the death certificate, if it was listed as an underlying and the most plausible cause of death, and if evidence of previous CHD was available. Similarly we used *ICD-8* codes 430–434 to define fatal stroke. In this analysis we also specifically considered deaths due to CVD (*ICD-8* codes 390.0-458.9 or 795.0-795.9).

### Statistical analysis

Individuals contributed person-time from the return of the baseline questionnaire in 1982 until the diagnosis of CVD, death, loss to follow-up, or the end of the follow-up period (June 30, 2012), whichever came first. To have reasonable number of cases in each category, the original 7 categories for bowel movement frequency were combined into 5 categories: more than once daily, daily, every 2 days, every 3–4 days, and every 5 days or less. Linear trend was assessed using mid-point values of each category of bowel movement frequency as continuous variables.

Cox proportional hazards estimated hazard ratio (HR) and 95% confidence interval (CI) for the association of bowel movement frequency with risk of incident CVD, total mortality, and cardiovascular mortality. No violation of proportional hazards assumption was observed. Multivariate models were used to minimize potential confounding, with covariates included based on biological relevance, clinical interest, strength of association with exposure or outcome, or percent change in the risk estimate (>5%). We adjusted for age, ethnicity, use of multivitamin, aspirin, other nonsteroidal anti-inflammatory drugs, thiazide diuretics, and thyroid hormone, family history of heart disease, baseline history of hypertension, hypercholesterolemia, or ulcerative colitis, cholecystectomy, menopausal status and use of menopausal hormone therapy, smoking pack-years, alcohol consumption, physical activity, dietary fiber intake, total energy intake, and dietary quality as assessed by Alternate Healthy Eating Index 2010 (AHEI-2010) score. We separately evaluated factors that could be potential mediators in the pathway between bowel movement frequency and cardiovascular risk, including BMI and diabetes. We used covariates assessed at baseline except for dietary factors, which were measured in 1980.

We performed sensitivity analyses by updating covariates to account for their variations over time. To further evaluate the robustness of our results, we also excluded cases in the first 4 years to reduce the potential for reverse causation. We conducted additional analyses by cross-classifying exposure with both infrequent bowel movement and laxative use.

We also assessed potential effect modification by baseline characteristics including age (median), BMI (≥30 kg/m^2^, 25–30 kg/m^2^, or < 25 kg/m^2^), smoking status (current smoker versus non-current smoker), alcohol intake (drinkers versus non-drinkers), levels of physical activity (median), baseline hypertension (hypertensive versus non-hypertensive), baseline diabetes (diabetic versus non-diabetic), laxative use (users versus non-users), dietary fiber intake (median), red meat intake (median), total energy intake (median), and AHEI score (median). We constructed cross-product terms between bowel movement frequency and these factors, with statistical significance of multiplicative interaction determined by using the likelihood ratio test.

Statistical analyses were conducted using SAS software version 9.3 (SAS Institute Inc). All p-values are 2-sided and a p-value of < 0.05 was considered as statistically significant.

## Additional Information

**How to cite this article**: Ma, W. *et al*. Associations of Bowel Movement Frequency with Risk of Cardiovascular Disease and Mortality among US Women. *Sci. Rep.* **6**, 33005; doi: 10.1038/srep33005 (2016).

## Supplementary Material

Supplementary Information

## Figures and Tables

**Table 1:** Baseline age-adjusted characteristics of participants by frequency of bowel movements in the Nurses’ Health Study (1982).

	Frequency of bowel movements				
	More than once per day(n = 8969)	Daily(n = 54264)	Every Other day(n = 15641)	Every 3–4 days(n = 6348)	Every 5 days or less(n = 1067)
Age, years*	49.0 (7.1)	48.6 (7.2)	47.0 (7.1)	47.0 (7.1)	47.3 (7.0)
Caucasian, %	98.0	97.6	97.3	97.1	97.0
Body mass index, kg/m^2^	26.4 (5.9)	24.6 (4.5)	24.2 (4.0)	24.1 (3.9)	24.0 (3.8)
Physical activity, h/week	2.5 (2.3)	2.4 (2.2)	2.3 (2.1)	2.1 (2.0)	2.2 (2.1)
Alcohol, g/d†	6.9 (12.2)	6.6 (10.7)	5.6 (9.1)	5.4 (9.1)	5.6 (9.7)
Premenopausal, %	45.1	48.4	47.9	45.3	44.7
Postmenopausal, never used hormone, %	30.6	30.3	30.0	31.0	31.5
Postmenopausal, past hormone user, %	10.6	9.5	9.9	11.3	11.9
Postmenopausal, current hormone user, %	13.8	11.8	12.2	12.4	11.9
Never smoking, %	43.8	43.3	46.2	44.9	44.8
Past smoking, %	29.1	28.5	30.5	30.4	29.1
Current smoking, %	27.0	28.0	23.1	24.4	26.1
Aspirin use, %	45.0	43.1	47.0	50.0	50.5
Other nonsteroidal anti-inflammatory drug use, %	6.9	4.7	4.9	5.1	7.1
Multivitamin use, %	44.3	41.0	39.1	36.0	36.5
Thiazide diuretics use%	16.8	12.7	12.5	12.6	13.8
Thyroid hormone use, %	12.1	9.2	8.2	7.4	7.9
Family history of myocardial infarction, %	20.4	19.0	18.1	19.3	19.5
Hypertension, %	25.4	18.2	16.5	16.0	15.8
Hypercholesterolemia, %	8.0	6.2	6.0	6.0	6.8
Diabetes, %	4.4	2.3	2.2	2.4	2.7
Ulcerative colitis, %	3.3	0.7	0.7	0.9	1.0
Cholecystectomy, %	13.3	6.8	7.1	7.7	8.0
Total energy intake, kcal/d†	1657.1 (524.0)	1571.1 (495.0)	1526.9 (491.9)	1513.3 (494.4)	1484.4 (516.5)
Total fiber intake (energy-adjusted), g/d†	16.2 (5.0)	16.4 (4.9)	16.4 (4.8)	16.1 (4.8)	16.2 (5.1)
Total red meat intake, serving/d†	1.5 (0.8)	1.4 (0.8)	1.4 (0.8)	1.4 (0.8)	1.4 (0.8)
Fruit intake, serving/d†	2.1 (1.5)	2.1 (1.4)	2.0 (1.4)	1.9 (1.3)	1.8 (1.3)
Vegetable intake, serving/d†	2.0 (1.2)	2.0 (1.1)	1.9 (1.1)	1.8 (1.1)	1.8 (1.2)
Coffee consumption, serving/d†	2.1 (2.0)	2.2 (1.9)	2.2 (1.9)	2.3 (2.0)	2.3 (2.0)
Alternate Healthy Eating Index score†	33.7 (9.2)	34.0 (8.9)	33.9 (8.6)	33.4 (8.5)	33.5 (8.6)
Laxative use weekly to daily, %	3.8	4.1	8.9	15.5	22.4

^*^Value is not age-adjusted.

^†^Dietary intakes were estimated with food-frequency questionnaire in 1980.

Values are means (SD) or percentages and are standardized to the age distribution of the study population.

**Table 2:** Relative risk (95% CI) of cardiovascular disease according to frequency of bowel movements in the Nurses’ Health Study (1982–2012)*.

	Frequency of bowel movements					P for trend†
	>1/Day	Daily	Every 2 days	Every 3–4 days	Every 5 days or less	
**Cardiovascular disease**						
Cases/person-years	1000/233698	4898/1457217	1163/430390	485 /173564	82/29053	
Model 1‡	1.25 (1.16, 1.33)	1.00 (reference)	0.91 (0.85, 0.97)	0.95 (0.86, 1.04)	0.93 (0.74, 1.15)	<0.001
Model 2§	1.15 (1.07, 1.23)	1.00 (reference)	0.94 (0.88, 1.01)	0.97 (0.88, 1.06)	0.94 (0.75, 1.17)	<0.001
Model 3||	1.13 (1.05, 1.21)	1.00 (reference)	0.94 (0.88, 1.00)	0.96 (0.87, 1.05)	0.93 (0.75, 1.16)	<0.001
Model 4#	1.05 (0.98, 1.13)	1.00 (reference)	0.96 (0.90, 1.02)	0.98 (0.90, 1.08)	0.96 (0.77, 1.19)	0.04
**Coronary heart disease**						
Cases/person-years	493/234078	2458/1459037	593/430801	235/173736	38/29086	
Model 1‡	1.22 (1.11, 1.34)	1.00 (reference)	0.92 (0.84, 1.00)	0.91 (0.80, 1.04)	0.85 (0.62, 1.17)	<0.001
Model 2§	1.12 (1.01, 1.23)	1.00 (reference)	0.96 (0.88, 1.05)	0.93 (0.81, 1.06)	0.86 (0.63, 1.19)	0.003
Model 3||	1.10 (1.00, 1.21)	1.00 (reference)	0.96 (0.87, 1.04)	0.91 (0.80, 1.04)	0.86 (0.62, 1.18)	0.004
Model 4#	1.00 (0.90, 1.10)	1.00 (reference)	0.98 (0.90, 1.07)	0.95 (0.83, 1.09)	0.89 (0.64, 1.22)	0.57
**Stroke**						
Cases/person-years	516/234029	2502/1458849	583/430814	256/173748	44/29077	
Model 1‡	1.26 (1.15, 1.39)	1.00 (reference)	0.90 (0.82, 0.98)	0.99 (0.87, 1.12)	0.98 (0.73, 1.32)	<0.001
Model 2§	1.16 (1.06, 1.28)	1.00 (reference)	0.93 (0.84, 1.01)	1.01 (0.88, 1.14)	0.99 (0.74, 1.34)	<0.001
Model 3||	1.15 (1.04, 1.27)	1.00 (reference)	0.93 (0.85, 1.02)	1.01 (0.88, 1.14)	0.99 (0.73, 1.34)	0.001
Model 4#	1.10 (1.00, 1.22)	1.00 (reference)	0.94 (0.85, 1.02)	1.02 (0.90, 1.16)	1.00 (0.74, 1.35)	0.03

^*^Cardiovascular disease was defined as coronary heart disease (symptomatic nonfatal myocardial infarction or fatal coronary heart disease) and stroke (nonfatal or fatal).

^†^P for trend was assessed by assigning the mid-point value in each category to participants and evaluating this as a continuous variable.

^‡^Adjusted for age.

^§^Further adjusted for ethnicity (Caucasian, yes/no), menopausal status (pre or postmenopausal (never, past, or current menopausal hormone use)), smoking status (never smoked, pack-years: 1–10, 10–24, 25–44, or ≥ 45), physical activity (hours/week: ≤ 1.0, 1.0–3.5, 3.6–6.0, or ≥ 6), family history of myocardial infarction (yes/no), baseline history of hypertension, hypercholesterolemia, or ulcerative colitis (yes/no), cholecystectomy (yes/no), and use of multivitamin, aspirin, other nonsteroidal anti-inflammatory drugs, thiazide diuretics, and thyroid hormone (yes/no).

^||^Further adjusted for alcohol intake (g/d: 0, 0.1–4.9, 5.0–14.9, 15.0–19.9, 20.0–29.9, or ≥ 30), Alternate Healthy Eating Index score (quintiles), dietary intake of total fiber (quintiles), and total energy intake (quintiles).

^#^Further adjusted for body mass index (kg/m^2^: < 23, 23–24.9, 25–26.9, 27–28.9, 29–30.9, 31–32.9, 33–34.9, 35–36.9, 37–38.9, 39–40.9, 41–42.9, 43–44.9, or ≥ 45) and baseline history of diabetes (yes/no).

**Table 3:** Relative risk (95% CI) of total and cardiovascular mortality according to frequency of bowel movements in the Nurses’ Health Study (1982–2012).

	Frequency of bowel movements					P for trend*
	> 1/Day	Daily	Every 2 days	Every 3–4 days	Every 5 days or less	
**Total mortality**						
Cases/person-years	2806/241676	13694/1495739	3078/439869	1264/177837	242/29735	
Model 1^†^	1.25 (1.20, 1.30)	1.00 (reference)	0.88 (0.85, 0.91)	0.90 (0.85, 0.95)	0.99 (0.87, 1.12)	< 0.001
Model 2^‡^	1.18 (1.14, 1.23)	1.00 (reference)	0.92 (0.88, 0.96)	0.92 (0.87, 0.97)	1.01 (0.89, 1.15)	< 0.001
Model 3^§^	1.17 (1.12, 1.22)	1.00 (reference)	0.92 (0.89, 0.96)	0.92 (0.87, 0.97)	1.01 (0.89, 1.15)	< 0.001
Model 4^||^	1.10 (1.06, 1.15)	1.00 (reference)	0.94 (0.90, 0.97)	0.94 (0.89, 0.99)	1.04 (0.91, 1.18)	< 0.001
**Cardiovascular mortality**						
Cases/person-years	644/243635	2955/1505636	604/442114	244/178752	44/29933	
Model 1^†^	1.32 (1.21, 1.44)	1.00 (reference)	0.83 (0.76, 0.91)	0.84 (0.74, 0.96)	0.86 (0.64, 1.15)	< 0.001
Model 2^‡^	1.19 (1.09, 1.30)	1.00 (reference)	0.88 (0.80, 0.96)	0.87 (0.76, 0.99)	0.89 (0.66, 1.20)	< 0.001
Model 3^§^	1.17 (1.07, 1.28)	1.00 (reference)	0.87 (0.80, 0.95)	0.86 (0.75, 0.98)	0.88 (0.65, 1.19)	< 0.001
Model 4^||^	1.05 (0.96, 1.15)	1.00 (reference)	0.90 (0.82, 0.98)	0.90 (0.79, 1.02)	0.92 (0.68, 1.24)	0.005

^*^P for trend was assessed by assigning the mid-point value in each category to participants and evaluating this as a continuous variable. Model adjustments were the same as shown in Table 2.

**Table 4:** Multivariable-adjusted relative risk of cardiovascular disease in relation to frequency of bowel movements according to baseline characteristics in the Nurses’ Health Study (1982–2012)*.

	Frequency of bowel movements					P for trend	P for interaction
	> 1/Day	Daily	Every 2 days	Every 3–4 days	Every 5 days or less		
Age							0.01
≥48 years (Case = 5759)	0.98 (0.91, 1.07)	1.00 (reference)	0.97 (0.90, 1.04)	0.93 (0.83, 1.04)	0.90 (0.69, 1.17)	0.45	
<48 years (Case = 1869)	1.25 (1.09, 1.43)	1.00 (reference)	0.94 (0.83, 1.07)	1.14 (0.96, 1.35)	1.08 (0.73, 1.61)	0.01	
Body mass index							0.18
≥30 kg/m^2^ (Case = 1413)	0.96 (0.84, 1.10)	1.00 (reference)	0.95 (0.80, 1.13)	1.08 (0.84, 1.39)	0.79 (0.41, 1.52)	0.72	
25–30 kg/m^2^ (Case = 2216)	1.20 (1.06, 1.36)	1.00 (reference)	1.04 (0.93, 1.17)	0.92 (0.76, 1.11)	1.06 (0.70, 1.59)	0.01	
<25 kg/m^2^ (Case = 5566)	1.03 (0.92, 1.15)	1.00 (reference)	0.92 (0.85, 1.01)	0.98 (0.87, 1.11)	0.95 (0.71, 1.26)	0.13	
Smoking status							0.47
Current smokers (Case = 2783)	1.00 (0.88, 1.12)	1.00 (reference)	0.98 (0.87, 1.09)	1.02 (0.87, 1.19)	1.10 (0.78, 1.54)	0.98	
Non-current smokers (Case = 4819)	1.08 (0.99, 1.18)	1.00 (reference)	0.96 (0.89, 1.04)	0.98 (0.87, 1.11)	0.88 (0.66, 1.17)	0.02	
Alcohol intake							0.12
Drinkers (Case = 5262)	1.05 (0.97, 1.15)	1.00 (reference)	0.93 (0.86, 1.01)	0.94 (0.83, 1.05)	1.11 (0.86, 1.42)	0.03	
Non-drinkers (Case = 2340)	1.04 (0.92, 1.18)	1.00 (reference)	1.03 (0.92, 1.15)	1.08 (0.91, 1.27)	0.68 (0.43, 1.05)	0.74	
Physical activity							0.37
≥1.5 h/week (Case = 3411)	1.07 (0.97, 1.19)	1.00 (reference)	0.96 (0.87, 1.06)	1.04 (0.90, 1.21)	1.20 (0.88, 1.65)	0.28	
<1.5 h/week (Case = 4124)	1.03 (0.94, 1.14)	1.00 (reference)	0.96 (0.88, 1.05)	0.94 (0.83, 1.06)	0.81 (0.59, 1.10)	0.09	
Hypertension							0.88
Hypertensive (Case = 2546)	1.10 (0.99, 1.23)	1.00 (reference)	0.96 (0.85, 1.08)	1.03 (0.86, 1.22)	1.01 (0.66, 1.52)	0.08	
Non-hypertensive (Case = 4989)	1.03 (0.94, 1.12)	1.00 (reference)	0.96 (0.89, 1.04)	0.96 (0.85, 1.07)	0.94 (0.73, 1.22)	0.18	
Diabetes							0.18
Diabetic (Case = 594)	0.97 (0.78, 1.21)	1.00 (reference)	1.02 (0.80, 1.31)	1.19 (0.84, 1.69)	1.29 (0.62, 2.71)	0.43	
Non-diabetic (Case = 6941)	1.06 (0.99, 1.14)	1.00 (reference)	0.96 (0.89, 1.02)	0.96 (0.87, 1.06)	0.93 (0.74, 1.18)	0.01	
Laxative use							0.14
Users (Case = 820)	1.23 (0.94, 1.62)	1.00 (reference)	0.92 (0.78, 1.09)	0.83 (0.68, 1.02)	0.74 (0.50, 1.11)	0.005	
Non-users (Case = 5317)	1.03 (0.95, 1.11)	1.00 (reference)	0.97 (0.89, 1.05)	1.10 (0.96, 1.26)	0.99 (0.68, 1.43)	0.55	
Total fiber intake							0.59
≥15.7 g/d (Case = 2347)	0.99 (0.87, 1.12)	1.00 (reference)	0.97 (0.86, 1.09)	0.90 (0.75, 1.09)	0.83 (0.53, 1.29)	0.55	
<15.7 g/d (Case = 2268)	1.06 (0.94, 1.20)	1.00 (reference)	0.94 (0.83, 1.07)	1.11 (0.94, 1.31)	0.83 (0.54, 1.28)	0.37	
Red meat intake							0.005
≥1.28 serving/d (Case = 2372)	0.88 (0.78, 1.00)	1.00 (reference)	0.88 (0.78, 1.00)	0.91 (0.76, 1.10)	0.75 (0.47, 1.17)	0.83	
<1.28 serving/d (Case = 2241)	1.20 (1.06, 1.35)	1.00 (reference)	1.05 (0.93, 1.18)	1.12 (0.94, 1.33)	0.93 (0.61, 1.42)	0.09	
Total energy intake							0.72
≥1509 kcal/d (Case = 2369)	0.97 (0.87, 1.10)	1.00 (reference)	0.98 (0.87, 1.11)	1.08 (0.90, 1.29)	0.85 (0.53, 1.35)	0.65	
<1509 kcal/d (Case = 2246)	1.08 (0.95, 1.23)	1.00 (reference)	0.92 (0.82, 1.04)	0.96 (0.80, 1.14)	0.81 (0.54, 1.22)	0.04	
Alternate Healthy Eating Index score							0.03
≥33.1 (Case = 2670)	1.15 (1.03, 1.28)	1.00 (reference)	1.00 (0.90, 1.12)	1.06 (0.90, 1.25)	0.87 (0.58, 1.30)	0.05	
<33.1 (Case = 2720)	0.92 (0.82, 1.04)	1.00 (reference)	0.87 (0.78, 0.98)	0.89 (0.75, 1.05)	0.72 (0.48, 1.09)	0.44	

^*^Results for age, physical activity, total fiber intake, red meat intake, total energy intake, and Alternate Healthy Eating Index score were stratified by the median value. Models were adjusted for the same covariates as shown in Model 4, Table 2.
