# Supplementary material for: Associations of Bowel Movement Frequency with Risk of Cardiovascular Disease and Mortality among US Women
Source: Sci Rep. 2016 Sep 6;6:33005. doi: 10.1038/srep33005 (PMC5011651; doi:10.1038/srep33005)
Supplement: Supplementary Information [file srep33005-s1.pdf]

Supplementary Material

Associations of Bowel Movement Frequency with Risk of Cardiovascular Disease and Mortality among US Women

Wenjie Ma, Yanping Li, Yoriko Heianza, Kyle D. Staller, Andrew T. Chan, Eric B. Rimm, Kathryn M. Rexrode, Lu Qi

Supplemental Table 1. Baseline age-adjusted characteristics of participants by frequency of bowel movements (daily versus non-daily) in the Nurses' Health Study (1982)

	Frequency of bowel movements		
	Daily (n=54264)	Non-daily (n=32025)	P-value*
Age, years†	48.6 (7.2)	47.6 (7.2)	<0.001
Caucasian, %	97.6	97.4	0.12
Body mass index, kg/m ²	24.6 (4.5)	24.8 (4.7)	<0.001
Physical activity, h/week	2.4 (2.2)	2.3 (2.1)	<0.001
Alcohol, g/d‡	6.6 (10.7)	5.9 (10.2)	<0.001
Premenopausal, %	48.4	46.6	<0.001
Postmenopausal, never used hormone, %	30.3	30.3	<0.001
Postmenopausal, past hormone user, %	9.5	10.5	0.31
Postmenopausal, current hormone user, %	11.8	12.7	0.02
Never smoking, %	43.3	45.2	<0.001
Past smoking, %	28.5	30.1	<0.001
Current smoking, %	28.0	24.5	<0.001
Aspirin use, %	43.1	47.0	<0.001
Other nonsteroidal anti-inflammatory drug use, %	4.7	5.6	<0.001
Multivitamin use, %	41.0	39.9	<0.001
Thiazide diuretics use%	12.7	13.8	0.09
Thyroid hormone use, %	9.2	9.2	0.16
Family history of myocardial infarction, %	19.0	19.1	0.52
Hypertension, %	18.2	19.0	0.39
Hypercholesterolemia, %	6.2	6.6	0.79
Diabetes, %	2.3	2.9	<0.001
Ulcerative colitis, %	0.7	1.5	<0.001
Cholecystectomy, %	6.8	9.0	<0.001

Total energy intake, kcal/d‡	1571.1 (495.0)	1559.6(505.9)	0.006
Total fiber intake (energy-adjusted), g/d‡	16.4 (4.9)	16.3(4.9)	<0.001
Total red meat intake, serving/d‡	1.4 (0.8)	1.4(0.8)	0.004
Fruit intake, serving/d‡	2.1 (1.4)	2.0(1.4)	<0.001
Vegetable intake, serving/d‡	2.0 (1.1)	1.9(1.1)	<0.001
Coffee consumption, serving/d‡	2.2 (1.9)	2.2(1.9)	<0.001
Alternate Healthy Eating Index score‡	34.0 (8.9)	33.7(8.8)	<0.001
Laxative use weekly to daily, %	4.1	9.1	<0.001

Values are means (SD) or percentages and are standardized to the age distribution of the study population.

* The P value was obtained by performing t-test for continuous variables and chi-square test for categorical variables.

† Value is not age-adjusted.

‡ Dietary intakes were estimated with food-frequency questionnaire in 1980.

Supplemental Table 2. Relative risk (95% CI) of non-daily bowel movements (more than once daily or less than once daily) in relation to key lifestyle and dietary factors in the Nurses' Health Study (1982)

	Multivariate relative risk (95% CI)*
Age, years	0.98 (0.98, 0.99)
Body mass index, kg/m ^{2}	1.00 (1.00, 1.00)
Physical activity, h/week	
≤1.0	1.00
1.0-3.5	0.93 (0.91, 0.95)
3.6-6.0	0.89 (0.87, 0.92)
≥6	0.90 (0.87, 0.94)
Alcohol, g/d	
0	1.00
0.1-4.9	0.99 (0.97, 1.01)
5.0-14.9	0.97 (0.94, 1.01)
15.0-19.9	0.93 (0.90, 0.96)
20.0-29.9	0.85 (0.82, 0.89)
≥30	0.90 (0.86, 0.95)
Pack-years, years	
0	1.00
1-10	1.00 (0.98, 1.03)
10-24	1.00 (0.98, 1.02)
25-44	0.92 (0.89, 0.94)
≥45	0.91 (0.88, 0.95)
Aspirin use	1.10 (1.08, 1.12)
Other nonsteroidal anti-inflammatory drug use	1.10 (1.06, 1.14)
Multivitamin use	0.97 (0.95, 0.98)
Thiazide diuretics use	1.05 (1.02, 1.08)
Thyroid hormone use	0.98 (0.95, 1.01)
Family history of myocardial infarction	1.00 (0.98, 1.02)
Hypertension	0.98 (0.96, 1.01)
Hypercholesterolemia	1.02 (0.98, 1.06)

Diabetes	1.12 (1.06, 1.17)
Ulcerative colitis	1.46 (1.38, 1.55)
Cholecystectomy	1.16 (1.13, 1.20)

*The multivariate relative risks were obtained from generalized linear models (PROC GENMOD) with Poisson distribution and log link, with adjustment for all variables in the table as well as ethnicity (Caucasian, yes/no), menopausal status (pre or postmenopausal (never, past, or current menopausal hormone use)), Alternate Healthy Eating Index score (quintiles), dietary intake of total fiber (quintiles), and total energy intake (quintiles). We did not use logistic regression because the outcome was not rare (prevalence of non-daily bowel movements: 37.1%) and the odds ratio would not be a good approximation of rate ratio.

Supplemental Table 3. Relative risk (95% CI) of cardiovascular disease, total mortality and cardiovascular mortality according to frequency of bowel movements in the Nurses' Health Study by excluding cases in the first 4 years (1982-2012)*

	Frequency of bowel movements					
	>1/Day	Daily	Every 2 days	Every 3-4 days	Every 5 days or less	P for trend†
**Cardiovascular disease**						
Cases/person-years	926/233544	4623/1456587	1113/430277	452/173488	78/29044	
Model 1‡	1.23 (1.14, 1.32)	1.00 (reference)	0.92 (0.86, 0.98)	0.93 (0.85, 1.03)	0.93 (0.74, 1.16)	<0.001
Model 2 §	1.13 (1.05, 1.21)	1.00 (reference)	0.95 (0.89, 1.02)	0.95 (0.86, 1.05)	0.94 (0.75, 1.18)	<0.001
Model 3‖	1.11 (1.04, 1.20)	1.00 (reference)	0.95 (0.89, 1.01)	0.94 (0.85, 1.04)	0.94 (0.75, 1.17)	<0.001
Model 4#	1.04 (0.96, 1.11)	1.00 (reference)	0.97 (0.90, 1.03)	0.97 (0.88, 1.07)	0.96 (0.77, 1.20)	0.10
**Total mortality**						
Cases/person-years	2758/241527	13524/1495217	3049/439779	1245/177780	241/29731	
Model 1‡	1.25 (1.20, 1.30)	1.00 (reference)	0.88 (0.85, 0.92)	0.90 (0.85, 0.95)	0.99 (0.87, 1.13)	<0.001
Model 2 §	1.18 (1.13, 1.23)	1.00 (reference)	0.92 (0.89, 0.96)	0.92 (0.86, 0.97)	1.02 (0.90, 1.16)	<0.001
Model 3‖	1.16 (1.12, 1.21)	1.00 (reference)	0.92 (0.89, 0.96)	0.92 (0.86, 0.97)	1.02 (0.90, 1.16)	<0.001
Model 4#	1.10 (1.05, 1.15)	1.00 (reference)	0.94 (0.90, 0.98)	0.94 (0.88, 0.99)	1.05 (0.92, 1.19)	<0.001
**Cardiovascular mortality**						
Cases/person-years	636/243453	2915/1505019	598/442007	240/178683	43/29929	
Model 1‡	1.32 (1.21, 1.44)	1.00 (reference)	0.84 (0.77, 0.91)	0.84 (0.74, 0.96)	0.85 (0.63, 1.14)	<0.001
Model 2 §	1.19 (1.09, 1.30)	1.00 (reference)	0.88 (0.80, 0.96)	0.86 (0.76, 0.98)	0.88 (0.65, 1.19)	<0.001
Model 3‖	1.17 (1.08, 1.28)	1.00 (reference)	0.87 (0.80, 0.95)	0.85 (0.75, 0.97)	0.87 (0.65, 1.18)	<0.001
Model 4#	1.06 (0.97, 1.15)	1.00 (reference)	0.90 (0.83, 0.99)	0.89 (0.78, 1.02)	0.91 (0.67, 1.23)	0.004

* Cardiovascular disease was defined as coronary heart disease (symptomatic nonfatal myocardial infarction or fatal coronary heart disease) and stroke (nonfatal or fatal).

† P for trend was assessed by assigning the mid-point value in each category to participants and evaluating this as a continuous variable.

‡ Adjusted for age.

§ Further adjusted for ethnicity (Caucasian, yes/no), menopausal status (pre or postmenopausal (never, past, or current menopausal hormone use)), smoking status (never smoked, pack-years: 1-10, 10-24, 25-44, or ≥45), physical activity (hours/week: ≤1.0, 1.0-3.5, 3.6-6.0, or ≥6), family history of myocardial infarction (yes/no), baseline history of hypertension, hypercholesterolemia, or ulcerative colitis (yes/no), cholecystectomy (yes/no), and use of multivitamin, aspirin, other nonsteroidal anti-inflammatory drugs, thiazide

diuretics, and thyroid hormone (yes/no).
|| Further adjusted for alcohol intake (g/d: 0, 0.1-4.9, 5.0-14.9, 15.0-19.9, 20.0-29.9, or ≥30), Alternate Healthy Eating Index score (quintiles), dietary intake of total fiber (quintiles), and total energy intake (quintiles).
Further adjusted for body mass index (kg/m^2: <23, 23-24.9, 25-26.9, 27-28.9, 29-30.9, 31-32.9, 33-34.9, 35-36.9, 37-38.9, 39-40.9, 41-42.9, 43-44.9, or ≥45) and baseline history of diabetes (yes/no).

Supplemental Table 4. Relative risk (95% CI) of cardiovascular disease, total mortality and cardiovascular mortality according to frequency of bowel movements in the Nurses' Health Study when adjusting for updated covariates (1982-2012)*

	Frequency of bowel movements					
	>1/Day	Daily	Every 2 days	Every 3-4 days	Every 5 days or less	P for trend†
**Cardiovascular disease**						
Cases/person-years	1000/233698	4898/1457217	1163/430390	485 /173564	82/29053	
Model 1‡	1.25 (1.16, 1.33)	1.00 (reference)	0.91 (0.85, 0.97)	0.95 (0.86, 1.04)	0.93 (0.74, 1.15)	<0.001
Model 2 §	1.20 (1.12, 1.28)	1.00 (reference)	0.95 (0.89, 1.01)	0.97 (0.88, 1.06)	0.94 (0.75, 1.16)	<0.001
Model 3‖	1.18 (1.10, 1.26)	1.00 (reference)	0.94 (0.88, 1.01)	0.95 (0.87, 1.05)	0.91 (0.73, 1.14)	<0.001
Model 4#	1.11 (1.04, 1.19)	1.00 (reference)	0.96 (0.90, 1.02)	0.98 (0.89, 1.07)	0.94 (0.75, 1.17)	<0.001
**Total mortality**						
Cases/person-years	2806/241676	13694/1495739	3078/439869	1264/177837	242/29735	
Model 1‡	1.25 (1.20, 1.30)	1.00 (reference)	0.88 (0.85, 0.91)	0.90 (0.85, 0.95)	0.99 (0.87, 1.12)	<0.001
Model 2 §	1.20 (1.16, 1.25)	1.00 (reference)	0.91 (0.88, 0.95)	0.92 (0.86, 0.97)	1.00 (0.88, 1.14)	<0.001
Model 3‖	1.18 (1.13, 1.23)	1.00 (reference)	0.91 (0.87, 0.94)	0.90 (0.85, 0.95)	0.97 (0.86, 1.10)	<0.001
Model 4#	1.16 (1.12, 1.21)	1.00 (reference)	0.91 (0.87, 0.94)	0.90 (0.85, 0.95)	0.97 (0.85, 1.10)	<0.001
**Cardiovascular mortality**						
Cases/person-years	644/243635	2955/1505636	604/442114	244/178752	44/29933	
Model 1‡	1.32 (1.21, 1.44)	1.00 (reference)	0.83 (0.76, 0.91)	0.84 (0.74, 0.96)	0.86 (0.64, 1.15)	<0.001
Model 2 §	1.27 (1.17, 1.38)	1.00 (reference)	0.88 (0.80, 0.96)	0.86 (0.76, 0.98)	0.88 (0.65, 1.19)	<0.001
Model 3‖	1.24 (1.14, 1.35)	1.00 (reference)	0.87 (0.79, 0.94)	0.84 (0.74, 0.96)	0.84 (0.62, 1.13)	<0.001
Model 4#	1.17 (1.08, 1.28)	1.00 (reference)	0.88 (0.80, 0.96)	0.86 (0.76, 0.98)	0.86 (0.63, 1.15)	<0.001

* Cardiovascular disease was defined as coronary heart disease (symptomatic nonfatal myocardial infarction or fatal coronary heart disease) and stroke (nonfatal or fatal).

† P for trend was assessed by assigning the mid-point value in each category to participants and evaluating this as a continuous variable.

‡ Adjusted for age.

§ Further adjusted for ethnicity (Caucasian, yes/no), menopausal status (pre or postmenopausal (never, past, or current menopausal hormone use)), smoking status (never smoker, past smoker, current smoker: 1-14, 15-24, or ≥25 cigarettes/d), physical activity (hours/week: ≤1.0, 1.0-3.5, 3.6-6.0, or ≥6), family history of myocardial infarction (yes/no), hypertension (yes/no), hypercholesterolemia (yes/no), ulcerative colitis or Crohn's disease (yes/no), cholecystectomy (yes/no), and use of multivitamin, aspirin, other nonsteroidal anti-inflammatory drugs, thiazide diuretics, and thyroid hormone (yes/no).

|| Further adjusted for alcohol intake (g/d: 0, 0.1-4.9, 5.0-14.9, 15.0-19.9, 20.0-29.9, or ≥30), Alternate Healthy Eating Index score (quintiles), dietary intake of total fiber (quintiles), and total energy intake (quintiles).

Further adjusted for body mass index (kg/m^2: <23, 23-24.9, 25-26.9, 27-28.9, 29-30.9, 31-32.9, 33-34.9, 35-36.9, 37-38.9, 39-40.9, 41-42.9, 43-44.9, or ≥45) and diabetes (yes/no).
